# COVID-19 Severity in Kidney Transplant Recipients According to Their Postvaccination Serological Assessment

**DOI:** 10.1016/j.ekir.2022.10.002

**Published:** 2022-10-11

**Authors:** Christophe Masset, Claire Garandeau, Aurélie Houzet, Delphine Kervella, Simon Ville, Diego Cantarovich, Alice Leclech, Claire Leman, Raphael Gaisne, Cécile Guillot-Gueguen, Océane Salomon, Clarisse Kerleau, Magali Giral, Jacques Dantal, Gilles Blancho, the Nantes DIVAT Consortium, Gilles Blancho, Julien Branchereau, Diego Cantarovich, Anne Cesbron, Agnès Chapelet, Jacques Dantal, Anne Devis, Florent Delbos, Clément Deltombe, Lucile Figueres, Raphael Gaisne, Claire Garandeau, Magali Giral, Caroline Gourraud-Vercel, Maryvonne Hourmant, Christine Kandel-Aznar, Georges Karam, Clarisse Kerleau, Delphine Kervella, Claire Leman, Alice Leclech, Christophe Masset, Aurélie Houzet-Meurette, Karine Renaudin, Simon Ville, Alexandre Walencik

**Affiliations:** 1 Institut de Transplantation Urologie Néphrologie (ITUN), CHU Nantes, Nantes, France; 2 Center for Research in Transplantation and Translational Immunology,Nantes Université, INSERM, UMR 1064, Nantes, France

**Keywords:** Covid-19, kidney transplant recipient, mRNA vaccine

## Introduction

Susceptibility of kidney transplant recipients (KTRs) to COVID-19 has been acutely apparent because of their increased risk of developing severe pneumonia^1^ and also, due to their lower vaccination response, conducing the transplant community to perform vaccine booster injections. Despite this, about 30% to 35% of patients remained seronegative after the third injection.^2^^,^^3^ Multiple reports highlighted the possibility of COVID-19 outbreak despite vaccination among KTRs,^4^ and the threshold of 264 BAU/ml antispike IgG has been initially determined to correlate with a protective neutralizing activity against symptomatic COVID-19 by the Alpha variant of concern (VOC).^5^ With the ongoing Omicron pandemic, many SARS-CoV-2 infections occur despite efficient vaccination due to the immune escape of this VOC.^6^ However, there is no current assessment of how COVID-19 severity relates to the postvaccination serological status of KTRs.

We investigated the outcomes of SARS-CoV-2 infection in KTRs depending on their vaccination status, their preinfection IgG antispike titer, administration of prophylactic monoclonal antibody and the VOC involved (Omicron or others).

## Results

### Description of the Cohort

Among the 352 patients followed-up in our institution who contracted COVID-19, 306 KTRs were retained in the final analysis as follows: 141 were not vaccinated (NO VAC), 45 were vaccinated without humoral response (SERO NEG), 44 were vaccinated with a weak humoral response (LOW POS), and 76 were vaccinated with a strong humoral response (HIGH POS) (Supplementary Figure S1). Complete methods of inclusion criterion, antibodies assays and groups definitions are described in the Supplementary Methods section. Specific treatments during COVID-19 and immunosuppression management among groups are summarized in Supplementary Table S1. The complete comparison between the groups’ characteristics is described in Table 1 and Supplementary Table S2.

**Table 1:** Description of the studied cohort

Patient's characteristics	All (*n* = 306)			NO VAC (*n* = 141)			SERO NEG (*n* = 45)			LOW POS (*n* = 44)			HIGH POS (*n* = 76)			*P*-value
	NA	n	%	NA	n	%	NA	n	%	NA	n	%	NA	n	%	
Male recipient	0	177	57.8	0	95	67.3	0	19	42.2	0	22	50.0	0	41	53.9	0.0100
Transplant rank ≥ 2	0	54	17.6	0	22	15.6	0	9	20.0	0	10	22.7	0	13	17.1	0.7117
Kidney transplant alone	0	272	88.8	0	123	87.2	0	43	95.5	0	40	90.9	0	66	86.8	0.4041
Deceased donor	3	260	85.8	1	123	87.8	0	39	86.6	0	35	79.5	2	63	85.1	0.7808
Calcineurin inhibitor treatment	0	264	86.2	0	120	85.1	0	39	86.6	0	43	97.7	0	62	81.5	0.0818
Belatacept treatment	0	14	4.5	0	6	4.2	0	3	6.6	0	0	0	0	5	6.5	0.3487
mTOR inhibitor treatment	0	27	8.8	0	13	9.2	0	2	4.4	0	2	4.5	0	10	13.1	0.2752
Antimetabolite treatment	0	229	74.8	0	103	73.0	0	35	77.7	0	35	79.5	0	56	73.6	0.7970
Steroid treatment	0	123	40.2	0	54	38.3	0	20	44.4	0	23	52.2	0	26	34.2	0.2259
Diabetes history	0	79	25.9	0	38	23.1	0	12	26.6	0	12	27.2	0	14	18.6	0.5253
Hypertension history	0	264	86.8	0	115	82.1	0	41	91.1	0	42	95.4	0	66	88.0	0.0826
Cardiovascular history	0	111	36.3	0	54	38.3	0	18	40.0	0	15	34.1	0	24	31.5	0.7207
RAAS blockers (ACEi or ARB)	0	102	33.3	0	52	36.8	0	11	24.4	0	18	40.9	0	21	27.6	0.1996
Respiratory history	1	51	16.7	0	30	21.2	1	16	35.5	1	8	18.1	1	9	12.0	0.0174
Neoplasia history	0	43	14.0	0	22	15.6	0	7	15.5	0	8	18.1	0	6	7.8	0.3377

	NA	Mean	SD	NA	Mean	SD	NA	Mean	SD	NA	Mean	SD	NA	Mean	SD	*P*-value
Recipient age (yr)	0	54.8	14.6	0	55.2	15.2	0	57.7	15.1	0	56.2	15.5	0	51.5	13.6	0.6474
Recipient BMI (kg.m^2^)	15	25.0	5.0	5	25.5	5.0	4	25.5	5.5	1	25.0	5.2	5	23.9	4.4	0.1490
Time from transplantation (yr)	0	8.2	7.8	0	8.7	7.9	0	5.8	6.3	0	6.5	6.2	0	9.7	8.9	0.0511
Baseline sera creatinemia (μmol/l)	7	144.4	70.6	6	145.0	73.1	0	161.0	91.6	0	147.9	63.6	1	131.3	52.3	0.1390

ACEi, angiotensin converting enzyme inhibitors; ARB, angiotensin receptor blockers; BMI, body mass index; HIGH POS, strong humoral response; LOW POS, weak humoral response; SERO NEG, vaccinated without humoral response; RAAS, renin angiotensin aldosterone system.

### Humoral Responses After SARS-CoV-2 Vaccination

**Figure 1:**
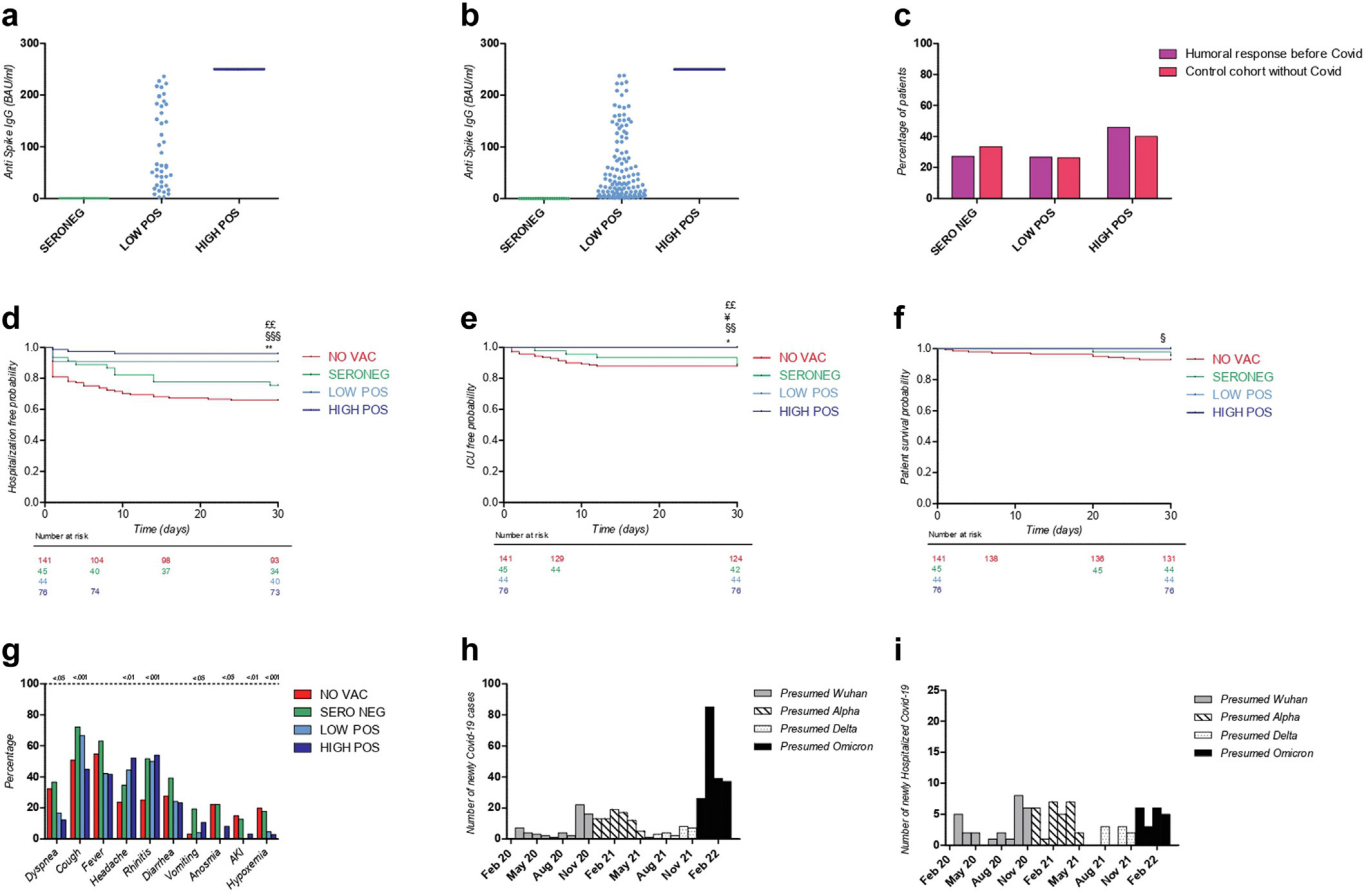
(a) Humoral response following SARS-CoV-2 vaccination in included COVID-19 patients from the 3 defined groups expressed by IgG antispike (BAU/ml). (b) Humoral response following SARS-Cov-2 vaccination in a cohort control of vaccinated patients (3 doses) without COVID-19 infection expressed by IgG antispike (BAU/ml). (c) Repartition of the patients depending on their postvaccination humoral response in the COVID-19 studied cohort and in the non-COVID-19 control cohort. (d) Survival without hospitalization depending on the vaccination status and the postvaccination serological assessment in the studied cohort. (e) Survival without intensive care unit hospitalization depending on the vaccination status and the postvaccination serological assessment in the studied cohort. (f) Survival without death depending on the vaccination status and the postvaccination serological assessment in the studied cohort. (g) Symptoms and major complications following COVID-19 depending on the vaccination status and the postvaccination serological assessment. (h) Representation of monthly new cases of COVID-19 in our center with the presumed different variants of concern based on the local epidemiology. (i) Representation of monthly new COVID-19 hospitalizations in our center with the presumed different variants of concern based on the local epidemiology. ∗represents a significant difference between NO VAC and LOW POS groups; ^§^represents a significant difference between NO VAC and HIGH POS groups; ^¥^represents a significant difference between SERO NEG and LOW POS groups; ^£^represents a significant difference between SERO NEG and HIGH POS groups; one symbol refers to a P-value < 0.05; 2 symbols to a P-value < 0.01 and 3 symbols to a P-value < 0.001. HIGH POS, strong humoral response; LOW POS, weak humoral response; SERO NEG, vaccinated without humoral response;

Of the KTRs, 72.8% developed a humoral response after vaccination. Almost all patients received an mRNA vaccine (1.8% received a heterologous vaccination). The serological assessment was performed 80 days (mean time) after the last injection, and SARS-CoV-2 infection was revealed 98 days (mean time) after the serological assessment. In the SERO NEG group, all patients except 2 had an undetectable humoral response; 2 had a detectable humoral response, though it was <1 BAU/ml. In the LOW POS group, the average humoral response level was 98 BAU/ml. In the HIGH POS group, all patients had a BAU titer >250/ml (greater than the laboratory’s threshold). In comparison, 477 KTRs who did not develop COVID-19 were vaccinated (3 doses) with available serological follow-up in our center. The overall seroconversion rate was 66.4%: 165 patients (33.5%) would be considered as SERO NEG, 125 as LOW POS (26.3%) and 192 as HIGH POS (40.2%) (Figure 1a–c**)**.

### COVID-19 Severity Depending on the Serological Status

Of the 306 included patients, 65 (21.2%)were hospitalized because of COVID-19: 48 in the NO VAC group (34.0% of the group); 10 in the SERO NEG group (22.2% of the group), 4 in the LOW POS group (9.1% of the group) and 3 in the HIGH POS group (3.9% of the group) (Figure 1d). These differences were significant between NO VAC and LOW POS (*P* = 0.0020), NO VAC and HIGH POS (*P* < 0.0001), SERO NEG and HIGH POS (*P* = 0.0006) and a trend between SERO NEG and LOW POS (*P* = 0.0636), Supplementary Table S3. Concerning intensive care unit admissions, they were significantly more frequent in the NO VAC group compared to the LOW POS group (*P* = 0.0175) and to the HIGH POS group (*P* = 0.0018), but also in the SERO NEG group compared to the LOW POS group (*P* = 0.0237) and to the HIGH POS group (*P* = 0.0030) (Figure 1e). Finally, patient death was more frequent in the NO VAC group compared to the HIGH POS group (*P* = 0.0183) (Figure 1f). The main symptoms and complications linked to SARS-CoV-2 infection are reported in Figure 1g. Mainly, patients vaccinated from the LOW POS and HIGH POS groups had a lower occurrence of dyspnea, anosmia, hypoxemia, and acute kidney injury.

Among the 45 patients in the SERO NEG group, 15 received MoAb prophylaxis, which seemed to lower the probability of hospitalization (Supplementary Figure S2a–c).

### Covid-19 Severity and the Variant Of Concern

A total of 166 patients presented a COVID-19 with a non-Omicron VOC and 140 patients were infected with the Omicron VOC (Supplementary Table S4−S7). Among patients with non-Omicron VOC, those from the HIGH POS group seemed to have a lower occurrence of severe COVID-19 forms (Supplementary Figure S3−S4). Among patients with Omicron, hospitalization and intensive care unit admission were higher in the SERO NEG group compared to the LOW POS and HIGH POS group (*P* = 0.0529 and *P* = 0.0075, respectively). Of note, the 9 patients from the NO VAC group were younger (49 years old vs. 57 years old) and with a lower body mass index (21.7 vs. 25.6) than patients from the SERO NEG group.

## Discussion

The results of our study confirm the significant benefit of SARS-CoV-2 vaccination in KTRs, leading to a lower rate of COVID-19 related hospitalizations, intensive care unit admissions and death. We demonstrated that a postvaccine humoral response, either high or low, drastically reduces occurrence of severe COVID-19 and death among KTR patients. Our data supports the clinical practice of routinely assessing the humoral response in KTR patients after SARS-CoV-2 vaccination in order to determine patients remaining at high-risk of severe COVID-19 despite vaccination.

It is important to note that the difference between unvaccinated and vaccinated KTRs differs depending on the study period and thus on the different VOCs (unvaccinated patients were mostly infected with non-Omicron VOC, whereas vaccinated patients were mostly infected with Omicron). This is linked to the low proportion of KTRs who remain unvaccinated in 2022 (mainly patients without other risk-factors for severe COVID-19 which are refractory to vaccine themselves), and to the demonstrated neutralizing activity of antispike IgG induced by vaccination against non-Omicron VOCs, thus reducing outbreak of COVID-19 in this population.^7^ However, our observed outcomes in postvaccination seronegative patients suggest that Omicron remained in at-risk patients without humoral response. The 9 unvaccinated patients who were infected with Omicron did not develop severe COVID-19, but their low number, added to their few associated risk factors (they were notably younger with lower body mass index) prevented any conclusion to be drawn. In KTRs without a humoral response after vaccination, administration of prophylactic monoclonal antibody seemed to reduce the occurrence of severe COVID-19, and thus may be proposed for nonresponders’ patients.

Our study is limited by several biases. First, we conducted a monocentric retrospective study that lacks the strength to perform a robust adjusted statistical analysis. Second, because VOC screening was not routinely performed by all of the laboratories, we had to extrapolate them from the local epidemiology. Finally, because serological screening was performed, on average, several weeks before COVID-19, we assumed that the accuracy of these results may have been modified. Indeed, antispike antibody titer slowly decreases over time,^8^ and this can explain why some KTRs with a high humoral response to the vaccine were infected with non-Omicron, and also, seronegative patients can convert several weeks later without any further injection.^9^

In conclusion, our study confirms the significant benefit of SARS-CoV-2 vaccination in KTRs and supports routine serological screening postvaccination in order to ensure the continued presence of antispike IgG. Indeed, patients without a humoral response remained at-risk of severe forms of COVID-19, and thus may benefit from monoclonal antibody prophylaxis, which seems to attenuate COVID-19 severity.

## Disclosure

All the authors declared no competing interests.

## Appendix

### Members of the Nantes DIVAT Consortium

#### Données Informatisées et VAlidées en Transplantation, DIVAT Cohort Collaborators (Medical Doctors, Surgeons, HLA Biologists)

Gilles Blancho, Julien Branchereau, Diego Cantarovich, Anne Cesbron, Agnès Chapelet, Jacques Dantal, Anne Devis, Florent Delbos, Clément Deltombe, Lucile Figueres, Raphael Gaisne, Claire Garandeau, Magali Giral, Caroline Gourraud-Vercel, Maryvonne Hourmant, Christine Kandel-Aznar, Georges Karam, Clarisse Kerleau, Delphine Kervella, Claire Leman, Alice Leclech, Christophe Masset, Aurélie Houzet-Meurette, Karine Renaudin, Simon Ville, Alexandre Walencik.

## Supplementary Material

**Supplementary Data**

**Figure S1.** Flowchart of the study

**Figure S2.** Survival without hospitalization for seronegative recipients depending on the prophylaxis by specific monoclonal antibodies

**Figure S3.** Survival without hospitalization depending on the vaccination status and the postvaccination serological assessment among different variants of concern

**Figure S4.** Symptoms and major complications depending on vaccination status and postvaccination serological assessment among different variants of concern

**Table S1.** Management of immunosuppressive drugs and specific anti SARS-CoV-2 therapy among studied patients

**Table S2.** Excluded patients versus all patients

**Table S3.** Successive *P*-values comparing the different cohorts in studied outcomes

**Table S4.** Description of the subcohort infected with presumed non-Omicron VOC

**Table S5.** Description of the vaccinated patients infected with presumed non-Omicron VOC depending on their postvaccine humoral response

**Table S6.** Description of the subcohort infected with presumed Omicron VOC

**Table S7.** Description of the vaccinated patients infected with presumed Omicron VOC depending on their postvaccine humoral response
