# Supplementary material for: COVID-19 Severity in Kidney Transplant Recipients According to Their Postvaccination Serological Assessment
Source: Kidney Int Rep. 2022 Oct 11;8(1):183–7. doi: 10.1016/j.ekir.2022.10.002 (PMC9550278; doi:10.1016/j.ekir.2022.10.002)
Supplement: Supplementary File (PDF) [file mmc1.pdf]

Supplementary Methods

Studied cohort

The included adult patients were kidney and/or pancreas transplant recipients followed in our institution with a functional allograft, who contracted Covid-19 between 1st March 2020 and 31st March 2022. Since the beginning of the Covid-19 pandemic, all patients followed-up in our center have been encouraged to declare to their transplant physician (by phone call and/or email through the website www.divat.fr) their SARS-Cov-2 infection status in order to facilitate close monitoring and eventually a hospitalization if required.

Assessment of post-vaccination serological status and Covid-19 severity

Since the beginning of the vaccination campaign, monitoring of the humoral response following vaccination has been routinely proposed for all of our patients. As this assessment was performed in multiple laboratories, several methods for detection of the anti-spike protein responses were used depending on laboratories' practices: chemiluminescent microparticle immunoassay (Abbott Architect®), chemiluminescence immunoassay (Siemens Atellica®), and electrochemiluminescence immunoassay (Roche Elecsys®). IgG anti-spike responses were considered positive if their level was above the laboratory threshold, and their respective Binding Antibody Units (BAU) were noted after conversion depending on the different laboratories. Depending on their vaccination status, and their anti-spike post-vaccination IgG assessment, patients were categorized as: non-vaccinated (NO VAC), vaccinated without humoral response (SERONEG), vaccinated with a low humoral response (LOW POS - BAU between 1 and 250/ml), and vaccinated with a high humoral response (HIGH POS - BAU > 250 BAU/ml;). All

serological assessments were considered before SARS-Cov-2 infection. If patients presented Covid-19 reinfection, only the first infection was considered as humoral response may have been impacted by a previous SARS-Cov-2 infection. Patients with missing data on their vaccination status and/or serological assessment post-vaccination were excluded from the analysis.

Similar methods were applied to analyze and compare post-vaccination (3 doses) humoral responses of a control cohort who did not presented Covid-19 infection.

SARS-Cov-2 infection was defined by the occurrence of RT-PCR positive for SARS-Cov-2 (nasal or broncho-alveolar) and/or positive nasal antigenic screening. Severe Covid-19 was defined by SARS-Cov-2 infection requiring hospitalization (traditional and/or ICU) and/or death during the 30 days following diagnosis. For the hospitalization status, we specifically noted the Covid-19 requiring hospitalization as opposed to hospitalizations associated with an asymptomatic Covid-19 or patients hospitalized daily for early treatments by specific MoAb and/or antiviral SARS-Cov-2 therapies. In cases of symptomatic Covid-19 hospitalizations occurring in patients already hospitalized (nosocomial transmission), the time between the diagnosis and the hospitalization was defined at one day.

Infection by the different variants of concern

When available, laboratories screening of VOCs was noted. However, in a majority of cases this screening was not routinely performed, and VOC status was extrapolated from our regional healthcare database when information was missing[21]. Briefly, SARS-Cov-2 infections that hadn't been screened for VOC and occurred after the 1st of January 2022 were considered as Omicron infections, whereas infections before

January 2022 were considered as non-Omicron infections (Wuhan, Alpha, and Delta).

Available Data

Baseline characteristics (age, comorbidities, allograft function) were noted as well as immunosuppressive therapies (maintenance therapy and their potent reduction during Covid-19). Covid-19 diagnosis and the delay between diagnosis and the last post-vaccination serological assessment were also described. Covid-19 symptoms were noted (dyspnea, cough, fever, rhinitis, diarrhea, anosmia), as well as the occurrence of complications such as Acute Kidney Injury (AKI) (defined by an increase ≥ 50% of sera creatininemia and/or requirement of dialysis) and hypoxemia (defined by the requirement of oxygen therapy). Covid-19 specific therapies such as administered MoAb and/or anti-viral therapy were also recorded.

Studied Outcomes

The main studied outcome was the occurrence of severe Covid-19 defined by SARS-Cov-2 infection requiring hospitalization (traditional and/or ICU) and/or death during the 30 days following diagnosis, depending on the vaccination status and the serological anti-spike IgG assessment post-vaccination. The secondary studied outcomes were ICU admission, death, and occurrence of severe Covid-19 by non-Omicron or Omicron VOC. Patients who did not get a severe Covid-19 were censored at 30 days after SARS-Cov-2 diagnosis.

Statistical Analysis

Continuous variables were expressed as mean or median, and categorical variables as total number (n) and percentage (%). For continuous variables, Student's t-tests or

Mann-Whitney tests were used; Chi-square tests or Fisher's exact tests were used for qualitative variables. One-way Anova or Kruskal-Wallis tests were used after verification of the normality distribution. Cumulative incidences of studied outcomes were analyzed using Kaplan-Meir curves. The significance threshold was set at 0.05 (two-tailed) and analyses were performed using GraphPad Prism version 5.0 (GraphPad Software, San Diego, CA, USA).

Ethical statement

Following informed consent, all patients' data were extracted from the DIVAT database (Données Informatisées et VAlidées en Transplantation; www.divat.fr, approved by the Comité National de l'Informatique et des Libertés CNIL, No.914184) and de-identified in order to respect confidentiality.

Supplementary Data

Table S1. Management of immunosuppressive drugs and specific anti SARS-Cov-2 therapy among studied patients

	NO VAC			SERONEG			LOW POS			HIGH POS		
	NA	n	%	NA	n	%	NA	n	%	NA	n	%
**Immunosuppression withdrawal**												
CNI	0	12	10.0	0	4	10.2	0	0	0	0	1	1.6
Antimetabolite	0	33	32.1	0	9	25.7	0	6	17.1	0	5	8.9
mTOR inhibitors	0	0	0	0	0	0	0	0	0	0	0	0
Belatacept	0	3	50.0	0	2	66.6	-	-	-	0	1	20.0
**Specific Covid-19 treatment**												
Dexamethasone	0	24	17.0	0	8	17.7	0	2	4.5	0	4	5.2
Tocilizumab	0	3	2.1	0	2	4.6	0	0	0	0	0	0
Remdesivir	0	3	2.1	0	1	2.3	0	0	0	0	2	2.6
Casivirimab + Imdevimab	0	7	4.9	0	8	18.2	0	5	11.3	0	0	0
Sotrovimab	0	1	0.7	0	5	11.1	0	3	6.8	0	0	0
Bamlanivimab + Etesevimab	0	0	0	0	0	0	0	1	2.2	0	0	0
Tixagevimab + Cilgavimab	0	0	0	0	0	0	0	0	0	0	1	1.3

Table S2 : Excluded patients vs all patients

	All patients (n = 352)			Studied patients (n = 306)			Excluded patients because of missing data (n = 46)			
	NA	n	%	NA	n	%	NA	n	%	**p-value**
**Male recipient**	0	202	57.3	0	177	57.8	0	25	54.3	0.7495
**Transplant rank ≥ 2**	0	62	17.6	0	54	17.6	0	8	17.3	1.0000
**Kidney transplant alone**	0	308	87.5	0	272	88.8	0	36	78.2	0.0544
**Deceased donor**	0	302	86.5	3	260	85.8	0	42	91.3	0.4851
**Calcineurin inhibitor treatment**	0	304	86.3	0	264	86.2	0	40	86.9	1.0000
**Belatacept treatment**	0	17	4.8	0	14	4.5	0	3	6.5	0.4747
**mTOR inhibitor treatment**	0	31	8.8	0	27	8.8	0	4	8.6	1.0000
**Antimetabolite treatment**	0	267	75.8	0	229	74.8	0	38	82.6	0.3549
**Steroid treatment**	0	137	38.9	0	123	40.2	0	14	30.4	0.2565
**Diabetes history**	0	97	27.6	0	79	25.9	0	18	39.1	0.0758
**Hypertension history**	0	306	87.4	0	264	86.8	0	42	91.3	0.4821
**Cardiovascular history**	0	125	35.5	0	111	36.3	0	14	30.4	0.5104
**RAAS blockers (ACE or ARA II)**	0	118	33.5	0	102	33.3	0	16	34.7	0.8678
**Respiratory history**	0	51	14.5	1	51	16.7	0	0	0	0.0006
**Neoplasia history**	0	46	13.0	0	43	14.0	0	3	6.5	0.2383
	NA	Mean	SD	NA	Mean	SD	NA	Mean	SD	**p-value**
**Recipient age (years)**	0	54.1	14.8	0	54.8	14.6	0	49.4	15.2	0.0295
**Recipient BMI (kg.m^2)**	20	25.1	5.0	15	25.0	5.0	5	25.6	5.0	0.5055
**Time from transplantation (years)**	0	8.2	7.8	0	8.2	7.8	0	8.0	7.3	0.9460
**Baseline sera creatinemia (µmol/l)**	8	144.7	70.7	7	144.4	70.6	1	146.2	72.0	0.9714

Table S3. Successive p-values comparing the different cohorts in studied outcomes

ALL

HOSPITALIZATION	NO VAC	SERONEG	LOW POS	HIGH POS
NO VAC		0.1962	0.0020	< 0.0001
SERO NEG	0.1962		0.0636	0.0006
LOW POS	0.0020	0.0636		0.2424
HIGH POS	< 0.0001	0.0006	0.2424	

ICU	NO VAC	SERONEG	LOW POS	HIGH POS
NO VAC		0.8040	0.0175	0.0018
SERO NEG	0.8040		0.0237	0.0030
LOW POS	0.0175	0.0237		1
HIGH POS	0.0018	0.0030	1	

DEATH	NO VAC	SERONEG	LOW POS	HIGH POS
NO VAC		0.4191	0.0725	0.0183
SERO NEG	0.4191		0.1596	0.0646
LOW POS	0.0725	0.1596		1
HIGH POS	0.0183	0.0646	1	

Non Omicron

HOSPITALIZATION	NO VAC	SERONEG	LOW POS	HIGH POS
NO VAC		0.6068	0.5331	0.1515
SERO NEG	0.6068		0.3882	0.3040
LOW POS	0.5331	0.3882		0.1250
HIGH POS	0.1515	0.3040	0.1250	

ICU	NO VAC	SERONEG	LOW POS	HIGH POS
NO VAC		0.9380	0.2653	0.2653
SERO NEG	0.9380		0.2810	0.2810
LOW POS	0.2653	0.2810		1
HIGH POS	0.2653	0.2810	1	

DEATH	NO VAC	SERONEG	LOW POS	HIGH POS
NO VAC		0.2624	0.7067	0.7067
SERO NEG	0.2624		1	1
LOW POS	0.7067	1		1
HIGH POS	0.7067	1	1	

Omicron

HOSPITALIZATION	NO VAC	SERONEG	LOW POS	HIGH POS
NO VAC		0.1520	1	0.2707
SERO NEG	0.1520		0.0048	0.0035
LOW POS	1	0.0048		0.3049
HIGH POS	0.2707	0.0035	0.3049	

ICU	NO VAC	SERONEG	LOW POS	HIGH POS
NO VAC		0.3244	1	1
SERO NEG	0.3244		0.0529	0.0075
LOW POS	1	0.0529		1
HIGH POS	1	0.0075	1	

DEATH	NO VAC	SERONEG	LOW POS	HIGH POS
NO VAC		0.3103	1	1
SERO NEG	0.3103		0.2719	0.1285
LOW POS	1	0.2719		1
HIGH POS	1	0.1285	1	

Table S4 : Description of the sub-cohort infected with presumed non-Omicron VOC

	All (n=166)			Non-vaccinated (n=132)			Vaccinated (n=34)			
	NA	n	%	NA	N	%	NA	n	%	**p-value**
**Male recipient**	0	105	63.2	0	87	65.9	0	18	52.9	0.1691
**Transplant rank ≥ 2**	0	30	18.1	0	21	15.9	0	9	26.4	0.2092
**Kidney transplant alone**	0	144	86.7	0	114	86.3	0	30	88.2	1.0000
**Deceased donor**	1	142	85.5	1	115	87.1	0	27	79.4	0.2770
**Calcineurin inhibitor treatment**	0	144	86.7	0	113	85.6	0	31	91.1	0.5721
**Belatacept treatment**	0	7	4.2	0	6	4.5	0	1	2.9	1.0000
**mTOR inhibitor treatment**	0	13	7.8	0	12	9.0	0	1	2.9	0.4715
**Antimetabolite treatment**	0	124	74.6	0	96	72.7	0	28	82.3	0.2788
**Steroid treatment**	0	68	40.9	0	50	37.8	0	18	52.9	0.1217
**Diabetes history**	0	50	30.1	0	41	31.0	0	9	26.4	0.6790
**Hypertension history**	0	141	85.4	0	108	82.4	0	33	97.0	0.0295
**Cardiovascular history**	0	66	39.7	0	51	38.6	0	15	44.1	0.5624
**RAAS blockers (ACE or ARA II)**	0	59	35.5	0	46	34.8	0	13	38.2	0.8410
**Respiratory history**	0	35	21.0	0	28	21.2	0	7	20.5	1.0000
**Neoplasia history**	0	30	18.0	0	22	16.6	0	8	23.5	0.4527
	NA	Mean	SD	NA	Mean	SD	NA	Mean	SD	**p-value**
**Recipient age (years)**	0	55.8	15.3	0	55.6	15.3	0	56.6	15.2	0.7233
**Recipient BMI ($kg.m^2$)**	7	25.5	5.0	5	25.8	5.0	2	24.5	4.8	0.2066
**Time from transplantation (years)**	0	8.3	7.8	0	8.6	7.8	0	7.2	7.8	0.1864
**Baseline sera creatinemia (µmol/l)**	6	142.7	70.1	5	144.7	74.5	1	135.1	49.8	0.4847

Table S5. Description of the vaccinated patients infected with presumed non-Omicron VOC depending on their post-vaccine humoral response

	SERONEG (n = 16)			LOW POS (n = 9)			HIGH POS (n = 9)			
	NA	n	%	NA	n	%	NA	n	%	**p-value**
**Male recipient**	0	7	56.2	0	4	44.4	0	5	55.5	0.8370
**Transplant rank ≥ 2**	0	5	31.2	0	3	33.3	0	1	11.1	0.4732
**Kidney transplant alone**	0	16	100	0	6	66.6	0	8	88.8	0.0457
**Deceased donor**	0	13	81.2	0	6	66.6	0	8	88.8	0.4912
**Calcineurin inhibitor treatment**	0	13	81.2	0	9	100	0	9	100	0.1571
**Belatacept treatment**	0	1	6.2	0	0	0	0	0	0	0.5602
**mTOR inhibitor treatment**	0	1	6.2	0	0	0	0	0	0	0.5602
**Antimetabolite treatment**	0	13	81.2	0	8	88.8	0	7	77.7	0.8156
**Steroid treatment**	0	9	56.2	0	5	55.5	0	4	44.4	0.8370
**Diabetes history**	0	4	25.0	0	3	33.3	0	2	22.2	0.8526
**Hypertension history**	0	16	100	0	8	88.8	0	9	100	0.2391
**Cardiovascular history**	0	9	56.2	0	3	33.3	0	3	33.3	0.4057
**RAAS blockers (ACE or ARA II)**	0	5	31.2	0	5	55.5	0	3	33.3	0.4572
**Respiratory history**	0	3	18.7	0	2	22.2	0	2	22.2	0.9693
**Neoplasia history**	0	5	31.2	0	2	22.2	0	1	11.1	0.5195
	NA	Mean	SD	NA	Mean	SD	NA	Mean	SD	**p-value**
**Recipient age (years)**	0	58.9	15.8	0	56.7	19.1	0	52.5	9.9	0.6183
**Recipient BMI (kg.m^2)**	1	25.4	4.3	1	23.2	5.7	0	24.3	4.8	0.5948
**Time from transplantation (years)**	0	7.3	8.8	0	7.1	7.1	0	7.3	7.7	0.9664
**Baseline sera creatinemia (µmol/l)**	0	152.7	49.6	0	108.0	39.6	1	130.2	50.8	0.0663

Table S6 : Description of the sub-cohort infected with presumed Omicron VOC

	All (n=140)			Non vaccinated (n=9)			Vaccinated (n=131)			
	NA	n	%	NA	n	%	NA	n	%	**p-value**
**Male recipient**	0	72	51.4	0	8	88.8	0	64	48.8	0.0338
**Transplant rank ≥ 2**	0	24	17.1	0	1	11.1	0	23	17.5	1.0000
**Kidney transplant alone**	0	128	91.4	0	9	100	0	119	90.8	0.3576
**Deceased donor**	2	118	85.5	0	8	88.8	2	110	85.2	1.0000
**Calcineurin inhibitor treatment**	0	120	85.7	0	7	77.7	0	113	86.2	0.6162
**Belatacept treatment**	0	7	5.0	0	0	0	0	7	5.3	1.0000
**mTOR inhibitor treatment**	0	14	10	0	1	11.1	0	13	9.9	1.0000
**Antimetabolite treatment**	0	105	75.0	0	7	77.7	0	98	74.8	1.0000
**Steroid treatment**	0	59	39.2	0	4	44.4	0	51	38.9	0.2167
**Diabetes history**	0	29	20.8	0	0	0	0	29	22.3	0.2038
**Hypertension history**	0	123	88.4	0	7	77.7	0	116	89.2	0.2997
**Cardiovascular history**	0	45	32.1	0	3	33.3	0	42	32.0	1.0000
**RAAS blockers (ACE or ARA II)**	0	43	30.7	0	6	66.6	0	37	28.2	0.0243
**Respiratory history**	1	16	11.5	0	2	22.2	1	14	10.7	0.2767
**Neoplasia history**	0	13	9.2	0	0	0	0	13	9.9	1.0000
	NA	Mean	SD	NA	Mean	SD	NA	Mean	SD	**p-value**
**Recipient age (years)**	0	53.6	13.8	0	49.2	11.3	0	53.9	13.9	0.3249
**Recipient BMI (kg.m^2)**	0	24.4	5.0	0	21.7	3.1	8	24.6	5.1	0.0939
**Time from transplantation (years)**	0	8.1	7.9	0	10.7	9.5	0	7.9	7.8	0.3026
**Baseline sera creatinemia (µmol/l)**	1	146.4	71.4	1	150.8	46.8	0	146.1	72.8	0.8577

Table S7. Description of the vaccinated patients infected with presumed Omicron VOC depending on their post-vaccine humoral response

	SERONEG (n = 29)			LOW POS (n = 35)			HIGH POS (n = 67)			
	NA	n	%	NA	n	%	NA	n	%	**p-value**
**Male recipient**	0	10	34.4	0	18	51.4	0	36	53.7	0.2093
**Transplant rank ≥ 2**	0	4	13.7	0	7	20.0	0	12	17.9	0.8050
**Kidney transplant alone**	0	27	93.1	0	34	97.1	0	58	86.5	0.1902
**Deceased donor**	0	26	89.6	0	29	82.8	2	55	82.0	0.7304
**Calcineurin inhibitor treatment**	0	26	89.6	0	34	97.1	0	53	79.1	0.0355
**Belatacept treatment**	0	2	6.8	0	0	0	0	5	7.4	0.2581
**mTOR inhibitor treatment**	0	1	3.4	0	2	5.7	0	10	14.9	0.0198
**Antimetabolite treatment**	0	22	75.8	0	27	77.1	0	49	73.1	0.8968
**Steroid treatment**	0	11	37.9	0	18	51.4	0	22	32.8	0.2920
**Diabetes history**	0	8	27.5	0	9	25.7	0	12	18.1	0.6204
**Hypertension history**	0	25	86.2	0	34	97.1	0	57	86.3	0.1734
**Cardiovascular history**	0	9	31.0	0	12	34.2	0	21	31.3	0.9468
**RAAS blockers (ACE or ARA II)**	0	6	20.6	0	13	37.1	0	18	26.8	0.3251
**Respiratory history**	0	5	17.2	0	5	14.2	1	4	6.0	0.1982
**Neoplasia history**	0	2	6.8	0	6	17.1	0	5	7.4	0.2476
	NA	Mean	SD	NA	Mean	SD	NA	Mean	SD	**p-value**
**Recipient age (years)**	0	57.0	14.9	0	56.0	12.1	0	51.4	14.1	0.1096
**Recipient BMI (kg.m^2)**	3	25.6	6.2	0	25.4	5.1	5	23.8	4.4	0.2163
**Time from transplantation (years)**	0	5.1	4.5	0	6.3	6.0	0	10.1	9.1	0.0381
**Baseline sera creatinemia (µmol/l)**	0	165.5	108.6	0	158.2	64.9	1	131.4	52.8	0.0285

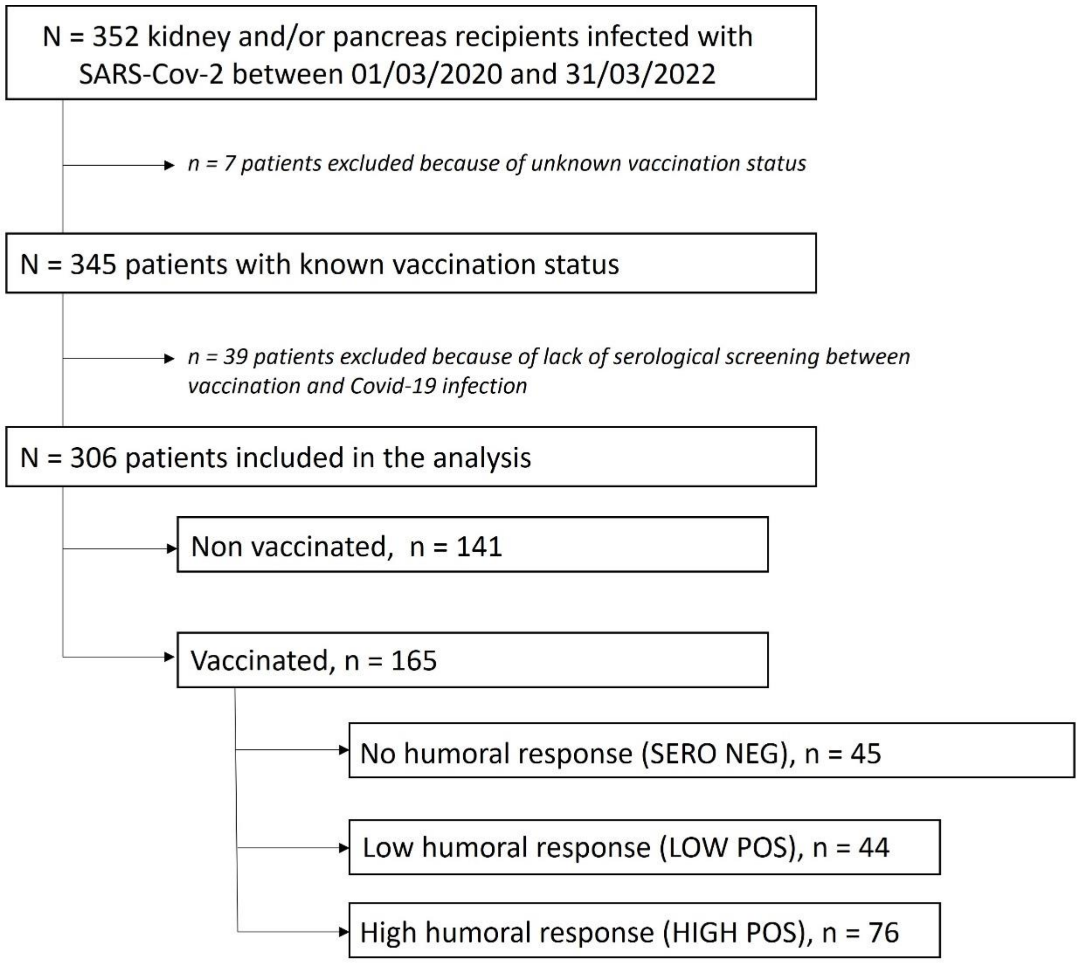

Figure S1 : Flowchart of the study

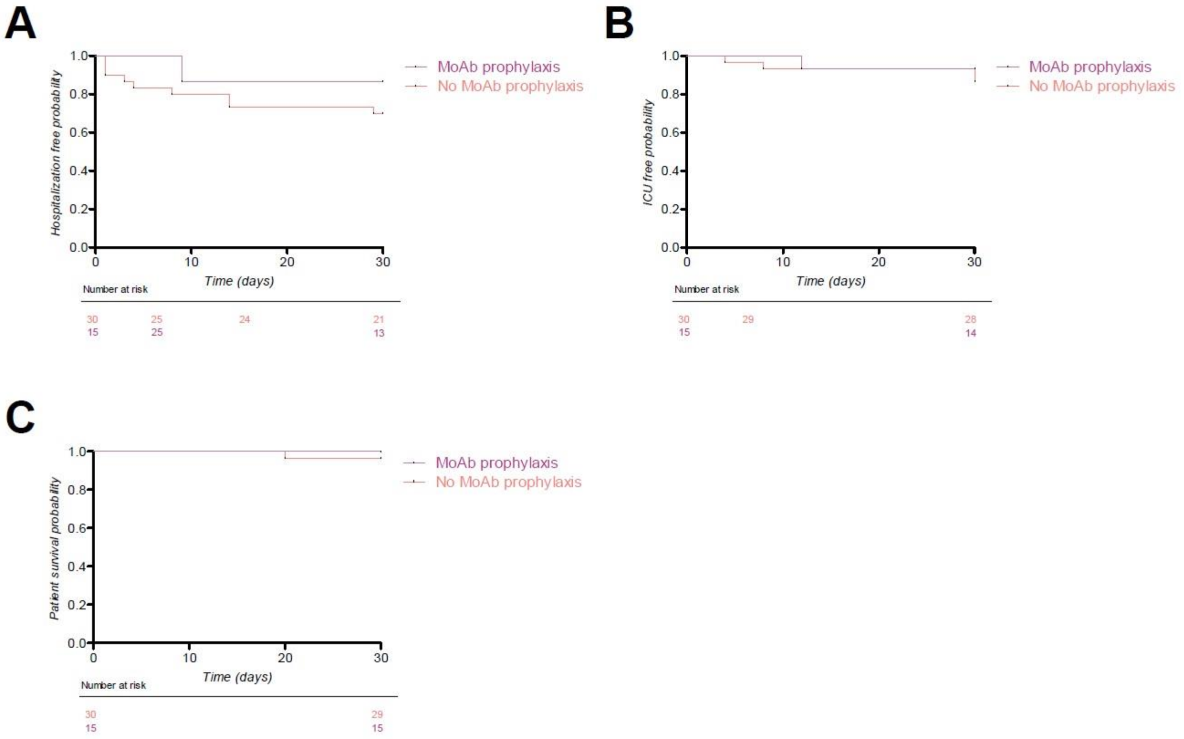

Figure S2. A. Survival without hospitalization for seronegative recipients depending on the prophylaxis by specific monoclonal antibodies. **B.** Survival without ICU hospitalization for seronegative recipients depending on the prophylaxis by specific monoclonal antibodies. **C.** Survival without death for seronegative recipients depending on the prophylaxis by specific monoclonal antibodies.

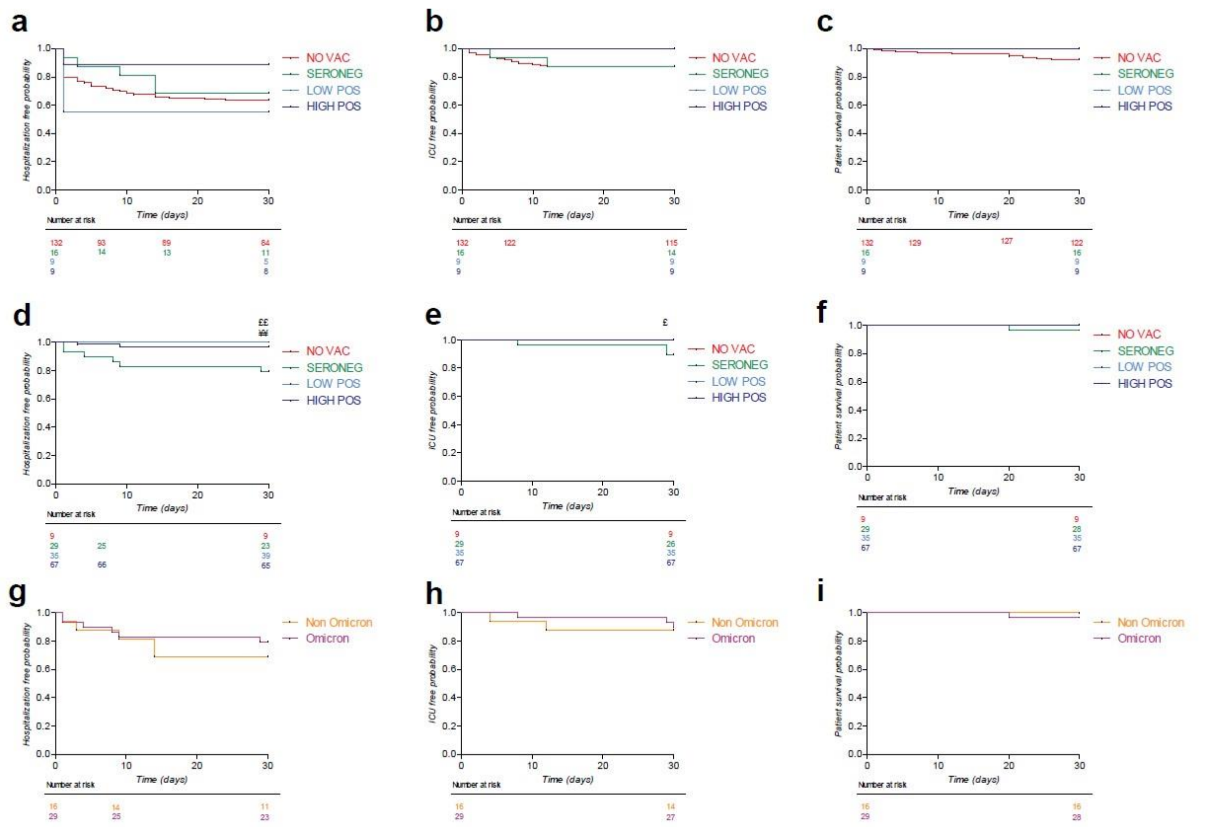

Figure S3. a. Survival without hospitalization depending on the vaccination status and the post-vaccination serological assessment during the non-Omicron pandemic. **b.** Survival without ICU hospitalization depending on vaccination status and post-vaccination serological assessment during the non-Omicron pandemic. **c.** Survival without death depending on vaccination status and post-vaccination serological assessment during the non-Omicron pandemic. **d.** Survival without hospitalization depending on the vaccination status and the post-vaccination serological assessment during the Omicron pandemic. **e.** Survival without ICU hospitalization depending on vaccination status and the post-vaccination serological assessment during the Omicron pandemic. **f.** Survival without death depending on vaccination status and post-vaccination serological assessment during the Omicron pandemic. **g.** Survival without hospitalization for seronegative patients depending on the presumed variant of concern (Omicron vs Non-Omicron). **h.** Survival without ICU hospitalization for seronegative patients depending on the presumed variant of concern (Omicron vs Non-Omicron). **i.** Patient survival for seronegative patients depending on the presumed variant of concern (Omicron vs Non-Omicron). ***** represents a significant difference between NO VAC and LOW POS groups; **§** represents a significant difference between NO VAC and HIGH POS groups; **¥** represents a significant difference between SERONEG and LOW POS groups; **£** represents a significant difference between SERONEG and HIGH POS groups; one symbol refers to a p-value < 0.05; two symbols to a p-value < 0.01 and three symbols to a p-value < 0.001.

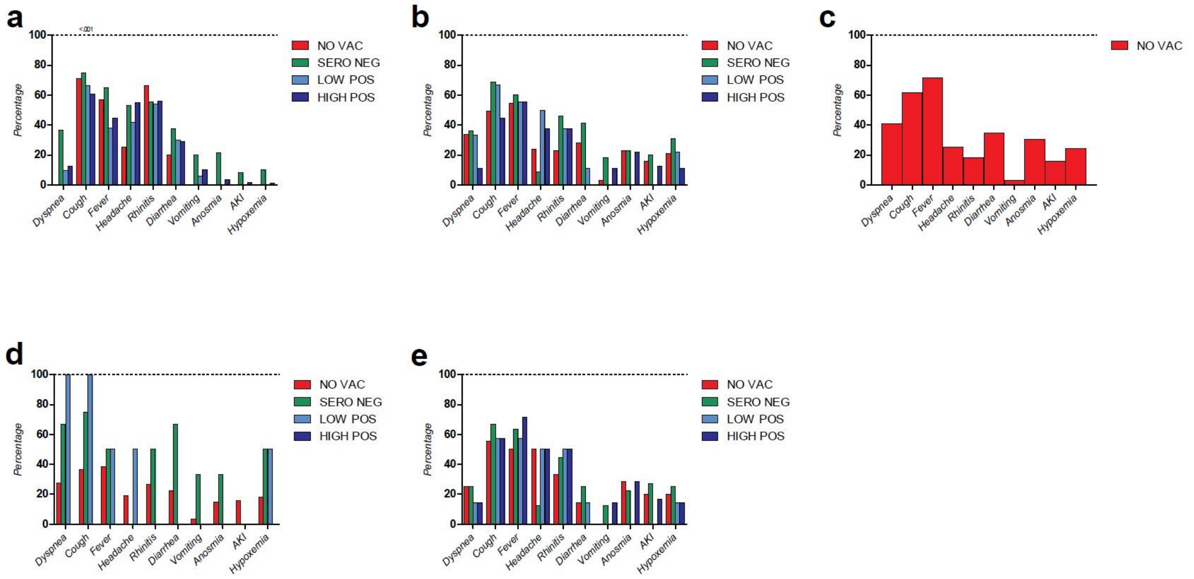

Figure S4. **a.** Symptoms and major complications following SARS-Cov-2 Omicron infection depending on vaccination status and post-vaccination serological assessment. **b.** Symptoms and major complications following SARS-Cov-2 Non-Omicron infection depending on vaccination status and post-vaccination serological assessment. **c.** Symptoms and major complications following SARS-Cov-2 Wuhan infection depending on vaccination status and post-vaccination serological assessment. **d.** Symptoms and major complications following SARS-Cov-2 Alpha infection depending on vaccination status and post-vaccination serological assessment. **e.** Symptoms and major complications following SARS-Cov-2 Alpha infection depending on vaccination status and post-vaccination serological assessment
